# Risk factors for femoral fracture in lateral decubitus direct anterior approach total hip arthroplasty using conventional stems: a retrospective analysis

**DOI:** 10.1186/s13018-021-02253-4

**Published:** 2021-01-30

**Authors:** Guanjun Sun, Yi Yin, Yongjie Ye, Qingshan Li

**Affiliations:** Suining Central Hospital, Suining, China

**Keywords:** Arthroplasty, Replacement, Hip, Direct anterior approach, Lateral decubitus position, Femur, Fracture

## Abstract

**Objective:**

To provide guidelines for surgery and reduce the incidence of fracture, this study analyzed the relationship between femoral fracture and related factors in direct anterior approach (DAA) total hip arthroplasty (THA) in the lateral decubitus position.

**Method:**

A retrospective series of 273 consecutive patients who underwent THA with the DAA in the lateral decubitus position was analyzed. Each surgery was performed by the same surgeon with a conventional operation bed and femoral stem. The correlations between the incidence of fracture and sex, age, body mass index (BMI), height, osteoporosis, the anterior superior iliac spine-greater trochanter distance (ASIS-GTD), and hip joint disease were analyzed by univariate analysis and logistic regression analysis.

**Results:**

Among all hip arthroplasty procedures, 35 hips had femoral fractures, including 30 greater trochanter fractures, 4 proximal femoral splits, and 1 femoral perforation. The incidence of fracture was 12.82%. Univariate analysis showed no significant difference in the incidence of fracture by sex, BMI, or age. However, osteoporosis caused an increase in the incidence of fracture, while the incidence of fracture decreased as height and the ASIS-GTD increased. The incidence of femoral neck fracture was lower in cases of osteonecrosis of the femoral head than in cases of other diseases. Logistic regression showed a significant correlation between osteoporosis, the ASIS-GTD, and fractures. Patients with osteoporosis had a high possibility of fracture (OR = 2.414); the possibility of fracture decreased with increasing ASIS-GTD (OR = 0.938).

**Conclusion:**

Lateral decubitus DAA THA can be successfully performed using a conventional operation bed and stem, effectively saving medical resources. Osteoporosis and a shorter ASIS-GTD were independent risk factors for femoral fracture.

## Introduction

Total hip arthroplasty (THA) is one of the most effective operations for the treatment of severe hip disease, and its efficacy has been unanimously recognized by physicians and patients. THA can effectively alleviate arthralgia, restore joint function, and correct articular malformation [1]. Multiple approaches can be used to perform THA, such as the posterior approach, direct lateral approach, and direct anterior approach (DAA). Surgeons may choose a specific approach according to their experience [2]. With the development of enhanced recovery after surgery (ERAS) in recent years [3, 4], the DAA has gained increasing attention, and its utilization in the clinic has increased. DAA is performed on the anterior hip joint through the interval between the tensor fascia lata and sartorius muscle [5]. DAA utilizes the neuromuscular interval and has the advantages of reduced intraoperative bleeding, a shorter invasive incision, and improved postoperative recovery [6]. However, the operative field in the DAA is restricted by the anterior superior iliac spine (ASIS) and greater trochanter (GT), especially on the femoral side, and the risk of femoral fracture is higher than that with the posterior approach [7]. Thus, the reliability of lateral DAA THA needs to be further verified [8].

In the USA, Europe, and some hospitals in China, DAA THA is performed in the supine position with a short femoral stem, which requires a special operation bed and is more expensive [9, 10]. There are no special operation beds in many hospitals in China, including that of the authors. All DAA THA procedures were performed using a conventional operation bed and conventional femoral stem in the lateral decubitus position, which had the benefits of a low cost, as ordinary equipment could be used. Thus, it is convenient to perform this operation [11–13]. To analyze the safety of this method for THA (lateral decubitus position, DAA, conventional femoral stem) and predict the risk of femoral fracture, we analyzed the correlation between the incidence of fracture and related risk factors, such as sex, age, BMI, height, osteoporosis, the ASIS-GTD, and hip diseases. This study also provides guidelines for preoperative planning to reduce the incidence of fracture.

## Material and methods

### Material

**Fig. 1:**
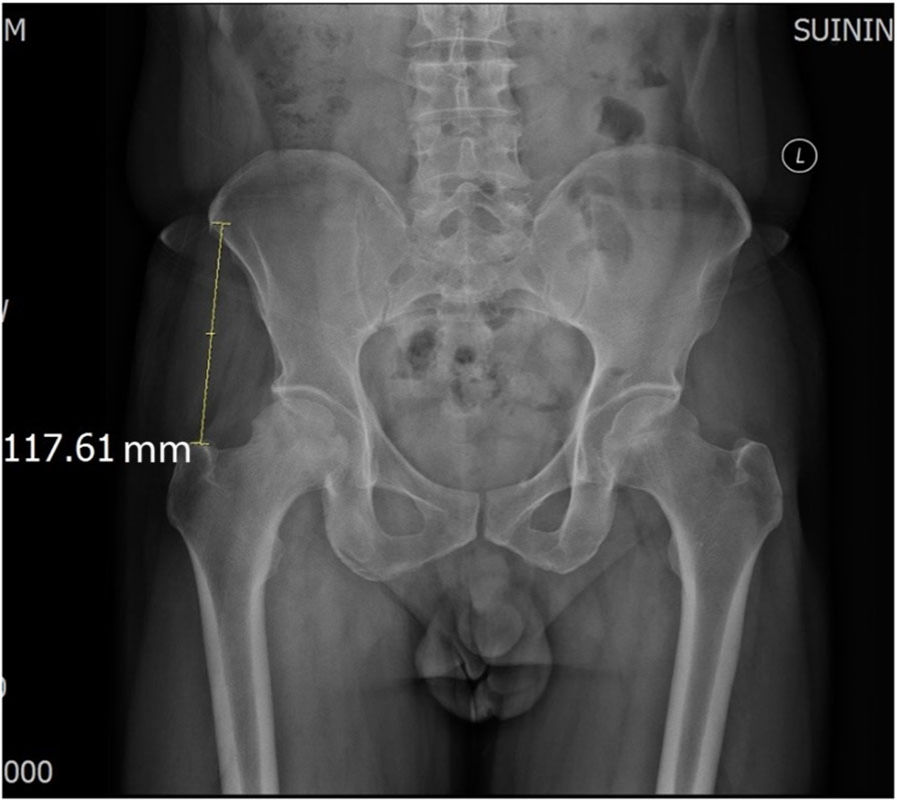
ASIS-GTD: the distance between the anterior superior iliac spine and greater trochanter, which was measured on standard supine pelvic X-rays

The consecutive analysis involved a total of 261 patients (273 hips) treated from January 2018 to January 2020 at Suining Central Hospital. All patients underwent DAA THA performed by the same surgeon, experienced in this surgery. There were 132 males (8 bilateral) and 129 females (4 bilateral), aged 33–89 years, with a BMI of 14.44–31.63 kg/m^2^. There were 88 patients with osteoporosis, which was measured by dual-energy X-ray absorptiometry (DXA). The diagnostic criterion was *T* ≤ − 2.5 SD (Table 1). The ASIS-GTD is the distance between the ASIS and GT. It was measured on supine pelvic X-rays using the PACS radiation software (Fig. 1). This study was approved by the ethics committee of the authors’ hospital. All patients signed written consent forms.

**Table 1:** Comparison of the data between the two groups [*n* (%),‾x ± s, P50 (P25, P75)]

Factor	No fractures (*n* = 238)	Fractures (*n* = 35)	*χ*^2^/*t*/*Z*	*P*
Sex			3.213^a^	0.073
Female	111	22		
Male	127	13		
Age	63.50 (51.00, 72.00)	63.00 (56.00, 75.00)	− 1.075^c^	0.283
Height (cm)	158.00 (153.00, 165.00)	153.00 (148.00, 160.00)	− 2.990^c^	0.003
Disease			13.408^a^	0.009
FNF	95	11		
ONFH	92	7		
OA	22	9		
DDH	17	5		
Others	12	3		
ASIS-GTD (mm)	101.76 ± 12.42	92.41 ± 13.14	4.127^b^	0.000
Osteoporosis			7.931^a^	0.005
No	166	16		
Yes	72	19		
BMI (kg/m^2^)	22.84 (21.08, 24.29)	22.83 (21.08, 24.24)	− 0.323^c^	0.746

^a^*χ*^2^ statistic

^b^*t* statistic

^c^*Z* statistic

The preoperative diagnosis included the following: osteonecrosis of the femoral head (ONFH, Ficat III, IV), osteoarthritis (OA), developmental dysplasia of the hip (DDH, Crowe I, II), femoral neck fracture (FNF), osteonecrosis of the femoral head after cannulated screw fixation of femoral neck fracture (internal fixation in the body, 12 patients), coxa plana, ankylosing spondylitis, and rheumatoid arthritis (RA). The femoral stems used were the Link LCU and Zimmer M/L, with the proximal 1/3 coated. The exclusion criteria were as follows: (1) systemic or local active infection, (2) severe malformation of the acetabulum or femur, and (3) hip ankylosis.

### Preoperative preparation

Before surgery, patients underwent standard pelvic anterior-posterior and ipsilateral femoral neck oblique radiography and CT of the hip joint. The femoral bone marrow diameter, femoral neck osteotomy position, and height of the hip rotation center were measured by X-ray, and the acetabular size and bone mass were measured by CT using the PACS radiation software. All these data were used to guide the surgery and improve the accuracy. Patients routinely received infusions of antibiotics and tranexamic acid half an hour before the operation.

### Surgical procedure

**Fig. 2:**
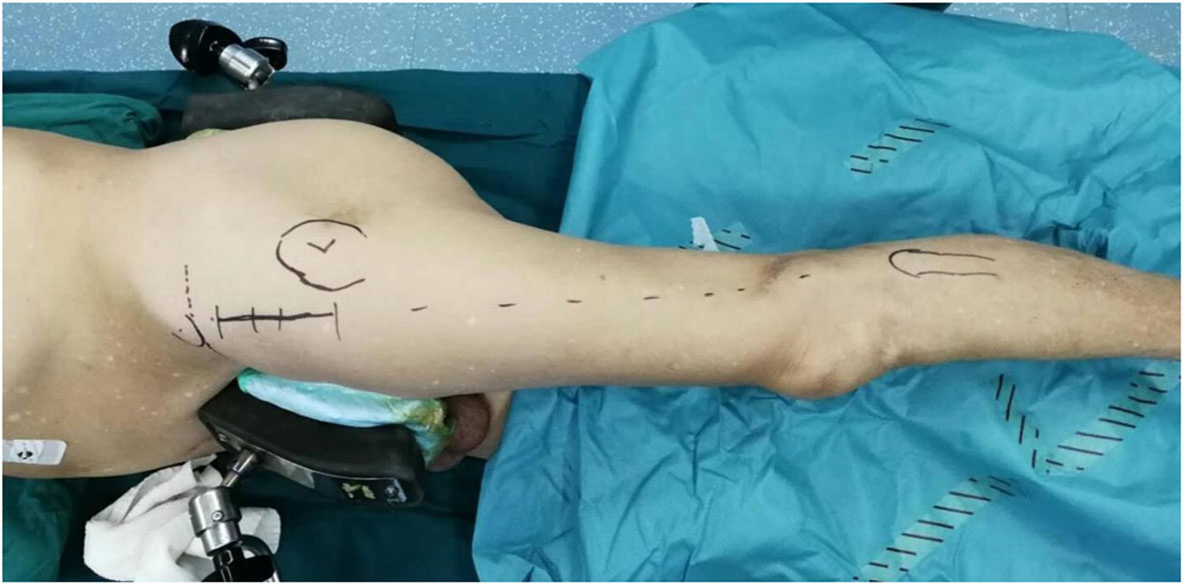
Approach: from 2 cm inferior and lateral of the anterior superior iliac spine (ASIS) to the fibular head, with a length of 8 cm

The patient lay on a regular operation bed in the lateral position to ensure that the horizontal axis of the pelvis was perpendicular to the bed (Fig. 2). An oblique incision was made originating 2 cm inferior and lateral to the ASIS inclined to the fibular head, with a length of 8 cm. The tensor fascia lata and sartorius muscle were divided, and then, the Heuter interval was entered. The ascending branch of the lateral circumflex femoral artery was cauterized and severed. A retractor was placed laterally to retract the tensor fascia lata, retract the rectus femoris muscle inward, and expose the anterior hip capsule. The anterior hip capsule was excised in an “L” shape. The anterior, superior, and inferior capsule was excised, and the femoral intertrochanteric line was exposed. Two retractors were placed, one superior and one inferior to the femoral neck. Femoral neck osteotomy was performed with a two-cut technique to remove the femoral head. The hip synovium, labrum, and residual capsule were removed. Three retractors were placed, one each along the anterior, superior, and inferior parts of the acetabulum, and then, the acetabulum and transverse ligament were exposed clearly. The acetabulum was then ground to an appropriate size. A corresponding liner was impacted at an abduction angle of 40–45° and anteversion angle of 15° or with reference to the implantation of the transverse acetabular ligament into the acetabular cup. The proximal femur was elevated, and the osteotomy stump was then loosened to an appropriate extent. The hip was adducted, extended, and externally rotated to expose the proximal femur. A retractor was placed at the rim of the greater trochanter, and a double-pronged retractor was placed superior to the lesser trochanter. The femoral neck was fixed, and then, the medullary cavity was expanded and ground to an appropriate size. A femoral stem was implanted, and femoral components were installed. The hip was reduced, and the muscle tension and joint stability were checked. Upon confirming a good position and appropriate length by C-arm fluoroscopy, the trial components were removed to place the femoral head prosthesis.

### Statistical methods

SPSS 21.0 was used to analyze the data. Count data are described by the number of cases (%). Differences between statistical inference groups were assessed by the χ^2^ test. Measurement data with a normal distribution are expressed as the mean ± standard deviation (x ± s), and group comparisons were performed by Student’s *t* test. Non-normally distributed measurement data are described by the median (quartile), and comparisons among groups were performed by the nonparametric rank-sum test. In the multivariate correlation analysis via a binary logistic regression model, the independent variables were significant factors evaluated by univariate analysis. The results are expressed by corrected odds ratios (ORs) and corresponding 95% confidence intervals (CIs). The results were considered to be significant at *p* < 0.05.

## Results

A total of 261 patients (273 hips) were included in the analysis; among these cases, there were 34 cases (35 hips) of femoral fracture, including 32 cases found during the operation and 2 cases found during the first postoperative X-ray examination. There were 30 greater trochanter fractures, 4 intertrochanteric and proximal femoral splits (fixation with steel wire, 1.46%, 4/273), and 1 femoral perforation. The total incidence of fracture was 12.82% (35/273), with 9.28% in males and 16.54% in females (*χ*^2^ = 3.213, *P* = 0.073). There was no significant difference in fracture rate by age or BMI (age: *Z* = − 1.075, *P* = 0.283; BMI: *Z* = − 0.323, *P* = 0.746). Osteoporosis was a risk factor for an increased fracture incidence (*χ*^2^ = 7.931, *P* = 0.005). There were significant differences in the fracture rate by height and ASIS-GTD (*Z* = − 2.990, *P* = 0.003; *t* = 4.127, *P* = 0.000). The data are shown in Table 1.

**Fig. 3:**
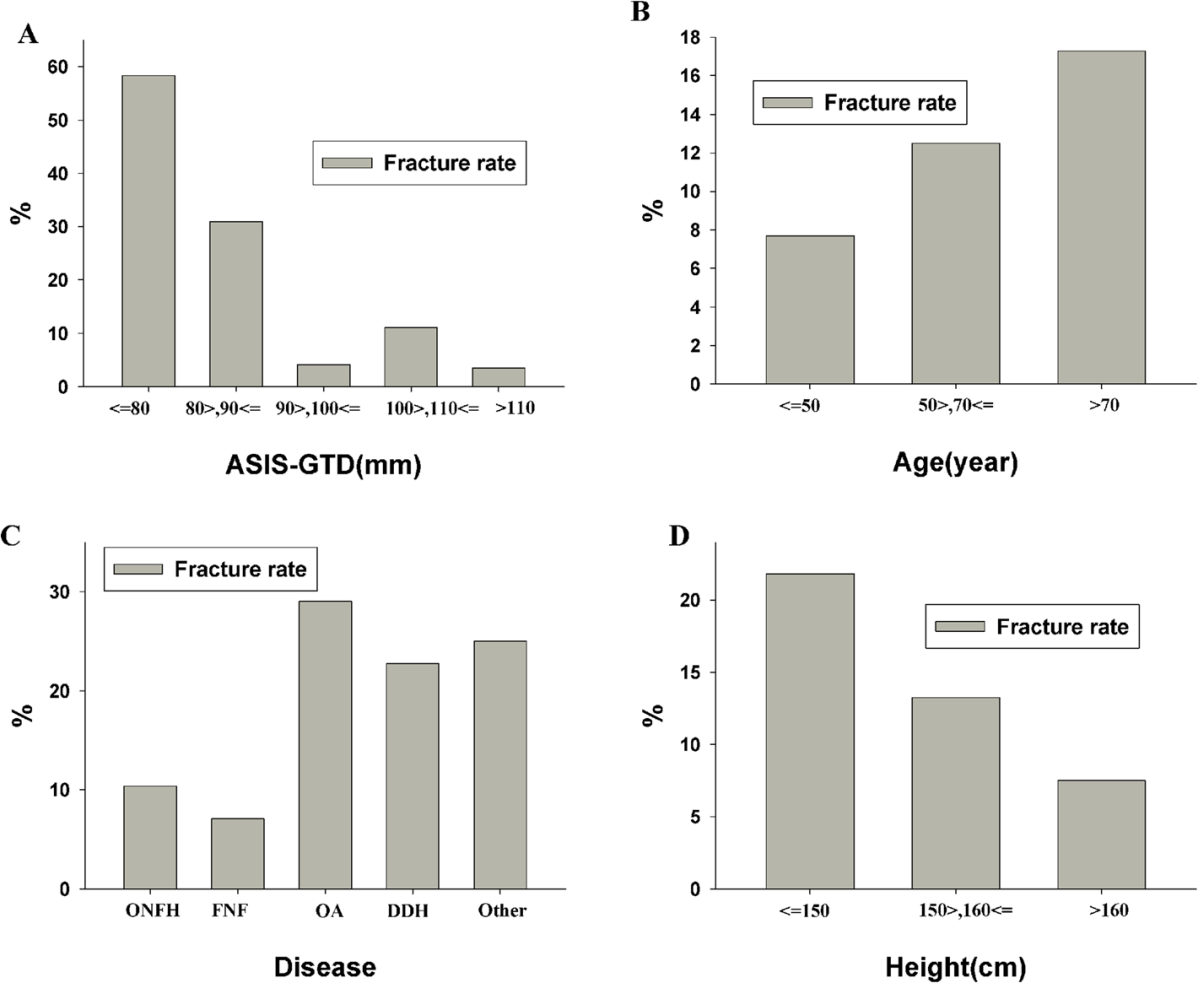
Correlation of fracture with the ASIS-GTD, age, disease, and height (univariate analysis). **a** As the ASIS-GTD increased, the fracture rate decreased gradually. When it was greater than 90 mm, the fracture rate decreased significantly (*P* < 0.05). **b** The fracture rate increased with age (*P* > 0.05). **c** The difference in fracture rate was statistically significant among different diseases (*P* < 0.05). **d** With increasing height, the fracture rate gradually decreased, and the difference was statistically significant (*P* < 0.05)

The fracture rate declined significantly when the patient height was greater than 160 cm (*χ*^2^ = 6.241, *P* = 0.0441). The fracture rate in the three height groups (≤ 150 cm, 150 ~ 160 cm, and > 160 cm) was 21.82%, 13.27% and 7.53%, respectively; there were no fractures in patients with a height of more than 170 cm. The fracture rate decreased with increasing ASIS-GTD, and when the ASIS-GTD was greater than 90 mm, the fracture rate decreased significantly (*χ*^2^ = 44.075, *P* = 0.000). The fracture rate in patients with an ASIS-GTD ≤ 80 mm, 80 ~ 90 mm, 90 ~ 100 mm, 100 ~ 110 mm, and > 110 mm was 58.3%, 30.95%, 4.17%, 11.11%, and 3.51%, respectively. The fracture rate varied by hip disease (*χ*^2^ = 13.408, *P* = 0.009), including femoral neck fracture (10.38%), osteonecrosis of the femoral head (7.61%), osteoarthritis (30%), DDH (23.81%), and others (rheumatoid arthritis, ankylosing spondylitis, and coxa plana, 16.67%) (Fig. 3).

In a binary logistic regression analysis, the fracture incidence was used as a dependent variable, and hip diseases, the ASIS-GTD, osteoporosis, and patient height were used as independent variables. The results showed that there was a significant correlation of the fracture incidence with osteoporosis and the ASIS-GTD (*P* < 0.05). Patients with osteoporosis had a high possibility of fracture, which was 2.414 times higher than that in patients without osteoporosis (OR = 2.414). An increasing ASIS-GTD reduced the possibility of fracture. For each additional unit (1 mm), the fracture incidence was diminished by 0.062 times (OR = 0.938). Hip diseases and patient height were not independent factors affecting the incidence of fracture (*P* > 0.05). The data are shown in Tables 2 and 3.

**Table 2:** Variable assignment table

Factor	Assignment
Fracture	
No	0
Yes	1
Osteoporosis	
No	0
Yes	1
ASIS-GTD	Continuous variables
Height	Continuous variables
Disease	
FNF	1
ONFH	2
OA	3
DDH	4
Others	5

**Table 3:** Logistic regression analysis of fracture

Factor	*β*	SE	Wald	OR	95% CI		*P*
					Lower limit	Upper limit	
Disease			5.103				0.277
FNF	Reference						
ONFH	− 0.298	0.596	0.250	0.742	0.231	2.388	0.617
OA	0.876	0.542	2.612	2.402	0.830	6.954	0.106
DDH	0.647	0.644	1.007	1.909	0.540	6.751	0.316
Others	− 0.088	0.797	0.012	0.915	0.192	4.361	0.912
Osteoporosis	0.881	0.432	4.166	2.414	1.036	5.627	**0.041**
ASIS-GTD	− 0.065	0.021	9.795	0.938	0.900	0.976	**0.002**
Height	− 0.006	0.030	0.034	0.994	0.937	1.055	0.854
Constant	4.695	4.228	1.233	109.447			0.267

*β* coefficient estimates, *Wald* Chi-square value, *OR* odds ratio, *CI* confidence interval.

## Discussion

With the development of ERAS in recent years, DAA has received increasing attention, and the utilization of DAA THA has increased because of its short invasive incision, reduced intraoperative bleeding, and improved recovery time. Many hospitals have carried out DAA THA and obtained satisfactory postoperative results [14]. With the increase in the number of operations, however, various intraoperative or postoperative complications have emerged [15]. The incidence of femoral fracture ranges from 0.1 to 22.4% [7, 16, 17]. In this retrospective study, all operations were carried out by an experienced surgeon. However, the incidence of femoral fracture was 12.82%. The fracture rate in the study might have a relationship with the extensive indications and use of a conventional stem. Many scholars have established strict selection criteria for DAA, including no articular malformations and obvious activity limitations [18]. In this study, all patients except those with hip ankylosis and severe deformity were selected for DAA surgery. Figure 3c shows that patients with DDH, OA, and other diseases (RA, coxa plana, and ankylosing spondylitis) who underwent DAA THA had a significantly higher fracture incidence than those with FNF and ONFH, indicating a certain influence of partial deformity and inflammatory diseases on the incidence of femoral fracture [19]. It should also be noted that patients with femoral neck fracture may have a high rate of osteoporosis but a normal hip anatomy, and the elderly have weak muscle strength, so the femoral fracture rate in these patients may be relatively low. In addition, the femoral stems used in this study were conventional stems, not short stems [20]. Conventional stems need more space than short stems, which may also have a certain impact on the incidence of fracture. Fortunately, only 4 hips (1.46%) with fracture required special treatment, indicating that lateral DAA THA could be successfully performed using conventional operation beds and conventional stems.

Some authors [15, 18] reported that patients with obesity and advanced age were not suitable for DAA THA, and the risk of complications such as fracture was high. In this study, the patient BMI was heterogeneous, and the continuity analysis did not show that BMI had a significant effect on the incidence of fracture. Indeed, the lateral decubitus position may facilitate exposure [21]. In the lateral decubitus position, the incision is at the highest point, and the peripheral tissues fall naturally away from the incision, so BMI (obesity) has no significant impact on the operation. In addition, compared to the supine position, the lateral position does not require a special surgical bed, which reduces medical costs and renders the procedure more convenient to perform. Figure 3b shows that the incidence of fracture increased with increasing age, but without a significant difference. However, the rate of osteoporosis increased with age [22]. Therefore, the real cause of the increase in the fracture incidence may be osteoporosis, not age or BMI [23]. Of course, it is well documented that in DAA, osteoporosis causes an increase in the fracture incidence [7], which was also confirmed by this study.

**Fig. 4:**
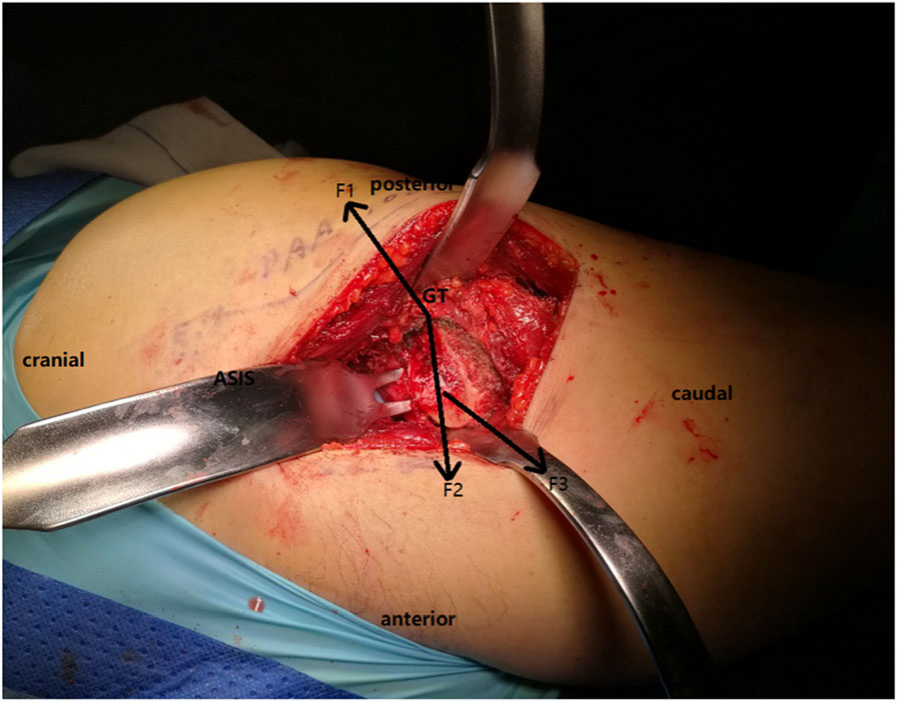
Schematic diagram of forces on proximal femur. F1: The traction force from the posterior superior muscles. F2: The direct force from the retractor. F3: The shear force from the femoral stem prosthesis

In DAA THA, operations involving the femur are difficult mainly due to the limited space between the anterior superior iliac spine and the greater trochanter. Under similar conditions, the smaller the ASIS-GTD is, the more difficult it is to expose the femur, leading to a higher incidence of fracture [24]. Compared with Europeans and Americans, Chinese individuals are relatively short [25, 26], which increases the difficulty of femoral exposure and the operation. Yang et al. [27] reported increasing difficulty performing the operation with shorter femoral necks. In this study, the ASIS-GTD was measured in the standard pelvic position, which was very simple. Both the univariate analysis and logistic regression analysis showed that the ASIS-GTD was an independent influencing factor for femoral fracture; the larger the ASIS-GTD was, the lower the incidence of fracture. This could be explained by mechanics. As shown in Fig. 4, the proximal femur was exposed, and the hip needed to be extended, adducted, and externally rotated. An acetabular retractor was placed lateral to the greater trochanter to facilitate exposure of the anterior femoral neck (F2). Except for the hip capsule, traction was established from posterior external rotation muscles and the posterior superior gluteus medius muscle (F1). The smaller the ASIS-GTD, the shorter the muscle between the two, and the more difficult it becomes to expose the femur. During the exposure, F1 and F2 were in opposite directions. F2 may cause greater trochanter fracture, and F1 may cause greater trochanter avulsion fracture. In the surgical procedure, if the femoral tools were blocked by the anterior superior iliac spine, an inward and downward shear force, F3, would be generated during femoral stem grinding and implanting processes, which could cause intertrochanter and proximal femoral fracture or posterior-inferior perforation. In contrast, the greater the ASIS-GTD, the lower the risks become. Of course, another force is needed to lift up the femur, which could be achieved by releasing the tensor fascia lata and the ischiofemoral ligament [28]. Figure 3a shows the stratified analysis, in which the fracture incidence dropped with increasing ASIS-GTD. The incidence of fracture tended to stabilize when the ASIS-GTD reached 90 mm. However, the fracture incidence fluctuated when the ASIS-GTD was between 100 mm and 110 mm. After analyzing the specific cases, the results showed that the fluctuation was related to the high proportion of patients with osteoarthritis and osteoporosis in the study.

In this study, 12 patients underwent bilateral DAA THA, including 8 patients with ONFH (ASIS-GTD ≥ 90 mm), 2 patients with RA (one ASIS-GTD = 76.22 mm), 1 patient with DDH (ASIS-GTD ≥ 90 mm), and 1 patient with OA (ASIS-GTD = 88.47 mm). However, only one patient suffered bilateral femoral fracture (RA, ASIS-GTD = 76.22 mm), indicating that the ASIS-GTD plays an important role in the incidence of fracture.

For exposure of the proximal femur, many scholars have reported different methods to prevent femoral fracture [29, 30]. After 3 years of practice, the authors have gained their own experience in releasing the proximal femur. Similar to the method Chughtai et al. reported [16], anterior, inferior, and superior capsule removal was performed in all patients using conventional methods to expose the greater trochanter fossa and femoral neck. In this way, we successfully completed the operation in some patients. Further release of the piriformis and posterior external rotary muscles was needed for patients in whom the surgery could still not be completed. In addition, for patients with a particularly short femoral neck, it was necessary to increase the osteotomy, which facilitated the exposure and adjustment of the lower limb length. With respect to the high-edge polyethylene liner, if the femur was particularly difficult to expose, the femur was processed first, and then, the liner and stem were installed.

### Limitations

Muscle relaxation after anesthesia influences the incidence of fracture. A deficiency of this study was the inconsistent anesthesia level, which may have had some impact on the results of the operation. Femoral exposure for DAA THA was more difficult in muscular patients [31]. The anesthesia level needs to be controlled in further research.

## Conclusion

In summary, lateral DAA THA could be successfully performed using a conventional operation bed and conventional stem, effectively saving medical resources. There was a significant correlation of the incidence of fracture with osteoporosis and the ASIS-GTD; fracture was more likely in patients with osteoporosis, and the possibility of fracture decreased with increasing ASIS-GTD. For high-risk patients, the surgeon should perform robust preoperative planning to reduce the incidence of fracture.

## Data Availability

The data and materials analyzed during the current study are available from the corresponding author on reasonable request.

## Abbreviations

DAA: Direct anterior approach
THA: Total hip arthroplasty
BMI: Body mass index
ASIS-GTD: Anterior superior iliac spine-greater trochanter distance
ERAS: Enhanced recovery after surgery
FNF: Femoral neck fracture
ONFH: Osteonecrosis of femoral head
DDH: Developmental dysplasia of the hip
OA: Osteoarthritis
RA: Rheumatoid arthritis
F(F_1_, F_2_, F_3_): Force
